# Ventricular Rupture due to Myocardial Infarction without Obstructive Coronary Artery Disease

**DOI:** 10.1155/2020/8847634

**Published:** 2020-11-09

**Authors:** Hendrik Lapp, Marcel Keßler, Thomas Rock, Franz X. Schmid, Dong-In Shin, Alexander Bufe, Heinrich G. Klues, Christian Blockhaus

**Affiliations:** ^1^Department of Cardiology, Heart Centre Niederrhein, Helios Clinics Krefeld, Germany; ^2^Department of Cardiothoracic Surgery, Heart Centre Niederrhein, Helios Clinics Krefeld, Germany; ^3^University Witten/Herdecke, Germany

## Abstract

An 87-year-old woman presenting with myocardial infarction and ST-segment elevation in the electrocardiogram suffered from pericardial effusion due to left ventricular rupture. After ruling out obstructive coronary artery disease and aortic dissection, she underwent cardiac surgery showing typical infarct-macerated myocardial tissue in situ. This case shows that even etiologically unclear and small-sized myocardial infarctions can cause life-threatening mechanical complications.

## 1. Introduction

Ischaemic heart disease is the most common cause of death worldwide. In Europe, acute myocardial infarction with ST-segment elevation (STEMI) has an incidence of 43 to 144 per 100.000 per year [1]. Compared to heart failure due to myocardial dysfunction or arrhythmic events, mechanical complications are less prevalent but nevertheless life-threatening and often associated with a lethal outcome. The rates of in-hospital mortality in patients with mechanical complications after myocardial infarction are described to be higher than 40%, highlighting ventricular septal defect, papillary muscle rupture, and complete ventricular free wall rupture as main complications [2]. In either case, rapid and efficient cardiothoracic surgery is the state-of-the-art medical treatment. Here, we describe the case of a patient with left ventricular wall rupture after acute myocardial infarction without obstructive coronary artery disease.

## 2. Case Report

**Figure 1:**
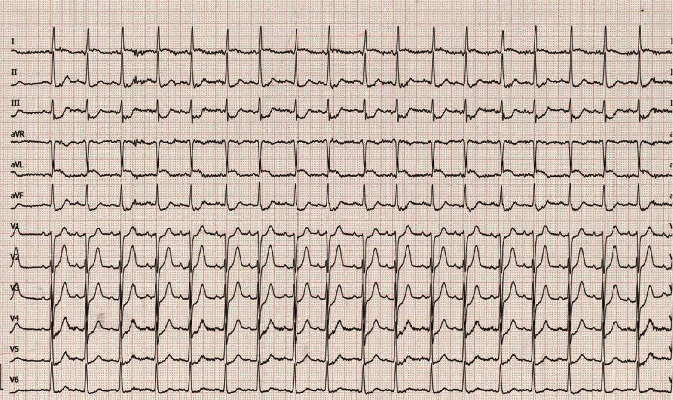
Initial ECG recording showing significant ST-segment elevations in leads I and aVL and corresponding ST-segment depressions in II, III, and aVF.

**Figure 2:**
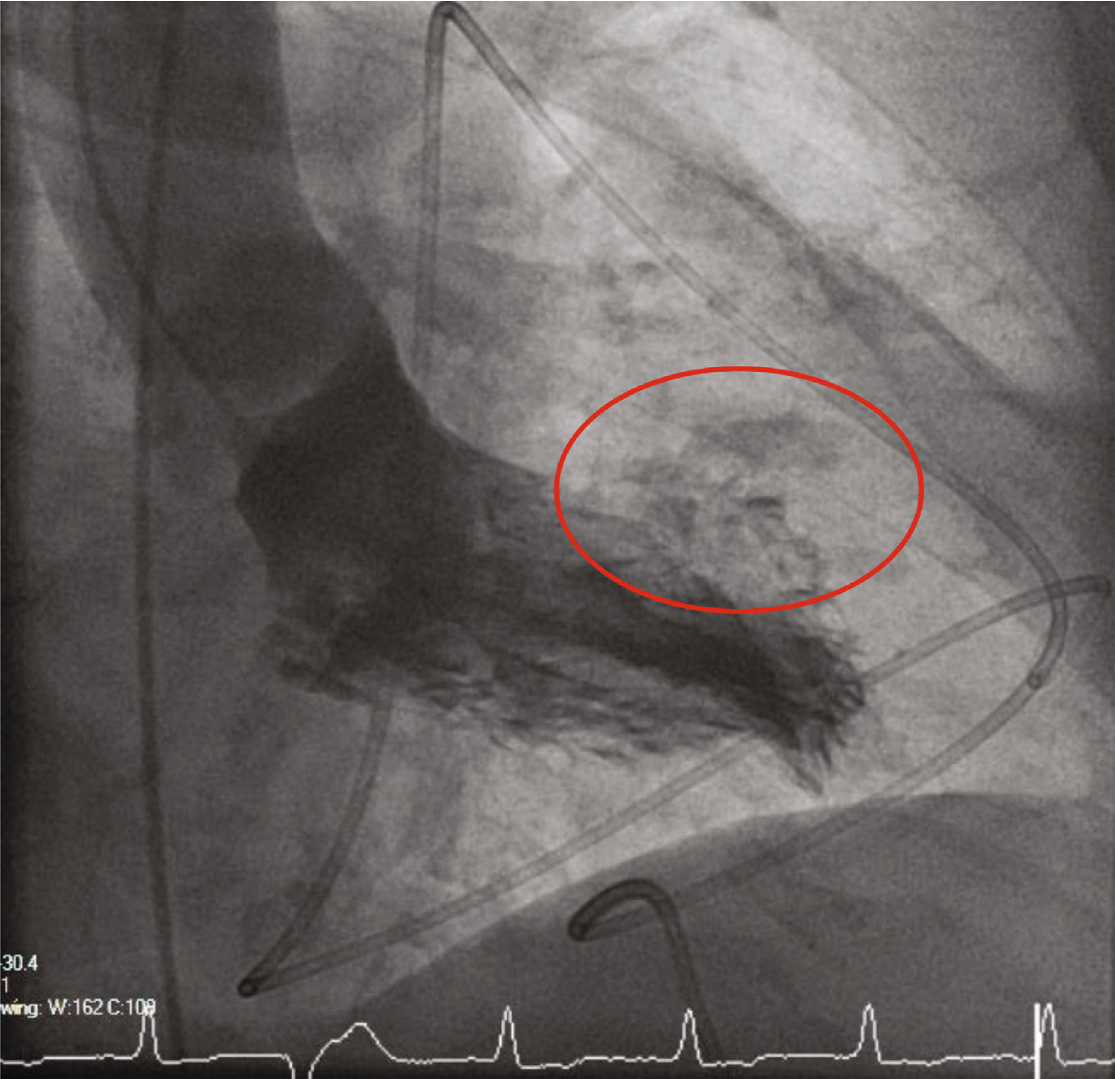
Laevocardiography showing evasion of contrast agent at the anterolateral ventricular wall.

An 87-year-old woman was transferred to our emergency department presenting with acute myocardial infarction. After syncope followed by acute chest pain in the morning during geriatric attendance in an external hospital, the immediately recorded electrocardiogram (ECG) showed significant ST-segment elevations in leads I and aVL and corresponding ST-segment depressions in all other leads, mainly II, III, and aVF (Figure 1). Upon arrival in our chest pain unit at 9:53 a.m., emergency physical examination revealed the woman to be suffering from dyspnea (oxygen saturation 96% while 10 l oxygen flow) at rest and severe cardiogenic shock with sinus tachycardia (117 bpm) and hypotension (blood pressure 82/50 mmHg). Varying doses of catecholamines (dobutamine/norepinephrine) were required to preserve a stable haemodynamic state. The patient was vigilant and fully orientated at all times. Emergency echocardiography revealed reduced left ventricular function, ventricular hypertrophy, and significant pericardial effusion. Based on the primary diagnostics and case history, aortic dissection type Stanford A was immediately excluded by computer tomography (CT). Subsequently, the patient was transferred to the cardiac catheterization laboratory. The levels of meanwhile analyzed high-sensitive Troponin T and creatine kinase were 1498 pg/ml and 92 U/l, respectively, indicating severe myocardial damage. Upon arrival in the cardiac catheterization laboratory, the pericardial effusion was instantaneously drained due to progressive haemodynamic instability. During further examination, the coronary arteries showed no signs of obstructive coronary artery disease, dissection, plaque rupture, or vasospasm. Invasive measurements demonstrated mid- to high-grade aortic stenosis. Laevocardiography revealed evasion of the contrast agent at the anterolateral ventricular wall due to ventricular rupture (Figure 2)—as already suspected in the initial computer tomography. After initial pericardiocentesis, the patient haemodynamic state showed a rapid deterioration, and increasing doses of vasopressors became necessary. Continuous pericardiocentesis with retransfusion of the blood over a 6F access in the femoral vein was necessary to obtain stable circulation followed by emergency transfer to our cardiothoracic surgery department for emergency surgery. After sternotomy and application of the heart-lung machine without complications, the left ventricular rupture in vivo showed signs of subsided myocardial infarction. The initially applied polypropylene sutures in the region of rupture were not sufficient due to macerated myocardial tissue after myocardial infarction. In a second attempt, the cardiac surgeons successfully used a bovine pericardial patch to cover the defect. After complex surgery taking several hours, active ventricular bleeding vanished and the patient was transferred to the cardiothoracic surgery intensive care unit. Subsequently, the patient demonstrated a dramatic and rapid increase of catecholaminergic substances without an adequate blood pressure response. On echocardiography, left ventricular function deteriorated finally leading to lethal cardiogenic shock within one hour after arrival at the cardiothoracic surgery intensive care unit.

## 3. Discussion

Ventricular rupture and overall mechanical complications after myocardial infarction are life-threatening events with high mortality. It occurs more frequently in elderly patients, females, or anterior infarctions often without previous symptoms of angina pectoris. There are three types of cardiac rupture described in literature [3]. Type I is an abrupt tear within the first 24 hours after myocardial infarction, type II is due to progressive myocardial damage, and type III is aneurysm-dependent wall thinning. Overall, the incidence declined significantly over the last decades with emerging primary coronary intervention [4]. Due to the rapid onset presenting with sudden hypotension, chest pain or cardiac murmurs after 2.6 days on average after acute myocardial infarction lethal outcomes remain still high [5]. Emergency echocardiography is the diagnostic tool of choice [1]. It is obvious that cardiothoracic surgery is the standard treatment, but powerful randomized controlled trials for best techniques and perioperative management are still missing. In smaller cohorts published by expert centers, it is suggested to cover the rupture site by autologous or synthetic pericardial patches in case of oozing bleedings, as well as the use of polypropylene sutures (direct suture techniques) in case of haemodynamically more challenging blowout bleedings [6]. Both state-of-the-art techniques were used in the presented case, but due to advanced macerated tissue, a sufficient cover of the rupture was challenging. Indeed, initial cardiac catheter examination exluded coronary artery disease and type I myocardial infarction [7] making spontaneous recanalization of plaque rupture unlikely, but there is evidence for myocardial infarction with nonobstructive coronary arteries (MINOCA) due to prolonged vasospasms, local thrombus formation, or embolization from other sites and for coronary microvascular dysfunction or obstruction potentially leading to small infarction size [8]. Only a few case reports of mechanical complications after myocardial infarction without coronary artery disease are published [9]. In the presented case, the exact underlying mechanism leading to myocardial infarction and subsequent cardiac rupture remains unclear; however, we suppose a temporary thrombotic occlusion is the most likely underlying pathology. Interestingly, in this case, emergency pericardiocentesis in the catheterization lab led to progressive haemodynamic deterioration. We suppose that an initial balance between LV hypertrophy, pericardial effusion, and tachycardia with autotamponading effect of the focal rupture in the hypertrophied myocardium may have occurred. This fragile haemodynamic stability was then abolished through the pericardiocentesis due to lowering external compression on the rupture site finally leading to progressive ventricular bleeding. In the literature, overall in-hospital survival rates between 38 and 78% are described [6], showing the current unpredictable influence of underlying pathology, patient-individual factors, comorbidities, time delay, and expertise of the center and the cardiothoracic surgeon.

## 4. Conclusion

At present, mechanical damages after myocardial infarction still remain a highly lethal complication. Ventricular rupture after MINOCA is a rare event but has to be kept in mind with the need for emergency, interdisciplinary and expert-based patient-focused individual treatment.
